# H_2_‐free Synthesis of Aromatic, Cyclic and Linear Oxygenates from CO_2_

**DOI:** 10.1002/cssc.201902340

**Published:** 2020-01-07

**Authors:** Laura Quintana Gomez, Amal K. Shehab, Ali Al‐Shathr, William Ingram, Mariia Konstantinova, Denis Cumming, James McGregor

**Affiliations:** ^1^ University of Sheffield Department of Chemical and Biological Engineering Mappin Street Sheffield S1 3JD UK; ^2^ BioEcoUVa Bioeconomy Institute Department of Chemical Engineering and Environmental Technology University of Valladolid 47011 Valladolid Spain

**Keywords:** carbon dioxide utilisation, high-temperature water (HTW), hydrothermal media, origin-of-life, phenol

## Abstract

The synthesis of oxygenate products, including cyclic ketones and phenol, from carbon dioxide and water in the absence of gas‐phase hydrogen has been demonstrated. The reaction takes place in subcritical conditions at 300 °C and pressure at room temperature of 25 barg. This is the first observation of the production of cyclic ketones by this route and represents a step towards the synthesis of valuable intermediates and products, including methanol, without relying on fossil sources or hydrogen, which carries a high carbon footprint in its production by conventional methods. Inspiration for these studies was taken directly from natural processes occurring in hydrothermal environments around ocean vents. Bulk iron and iron oxides were investigated to provide a benchmark for further studies, whereas reactions over alumina and zeolite‐based catalysts were employed to demonstrate, for the first time, the ability to use catalyst properties such as acidity and pore size to direct the reaction towards specific products. Bulk iron and iron oxides produced methanol as the major product in concentrations of approximately 2–3 mmol L^−1^. By limiting the hydrogen availability through increasing the initial CO_2_/H_2_O ratio the reaction could be directed to yield phenol. Alumina and zeolites were both observed to enhance the production of longer‐chained species (up to C_8_), likely owing to the role of acid sites in catalysing rapid oligomerisation reactions. Notably, zeolite‐based catalysts promoted the formation of cyclic ketones. These proof‐of‐concept studies show the potential of this process to contribute to sustainable development through either targeting methanol production as part of a “methanol economy” or longer‐chained species including phenol and cyclic ketones.

## Graphical abstract

**No H_2_ required**: The synthesis of oxygenate products, including cyclic ketones and phenol, from carbon dioxide and water in the absence of gas‐phase hydrogen is demonstrated. The reaction takes place in subcritical conditions at 300 °C and pressure at room temperature of 25 barg. Alumina and zeolite‐based catalysts demonstrate, for the first time, the ability to use acidity and pore size to direct the reaction towards specific products.

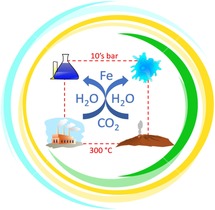

## Introduction

Industrial chemicals and fuels are ubiquitous in modern society. Their production from hydrocarbons, oxygenates, cyclic and aromatic compounds is essential for the global economy; however, an overwhelming proportion are produced from unsustainable sources. Sustainable development is defined as “development that meets the needs of the present without compromising the ability of future generations to meet their own needs”,^1^ and hence new methods of chemical production are essential to meet this goal. According to recent estimates, over 500 Mt yr^−1^ of fossil reserves are consumed by the chemical industry in the synthesis of necessary platform chemicals such as methanol, olefins and aromatics.^2^ Consequently, the chemical sector is the third largest industrial emitter of carbon dioxide (CO_2_) and a major contributor to anthropogenic climate change.^3^ This has stimulated investigations into carbon capture from power and chemical plants,^4^ for example, as sodium bicarbonate (NaHCO_3_).^5^, ^6^ In this context, the direct conversion of CO_2_ to fuel and organic molecules presents an opportunity to utilise an abundant waste resource. Many methods of CO_2_ conversion have been reported including hydrogenation, photochemical and electrochemical reduction,^7^ but low yields and/or high costs remain a major hindrance to adoption of these technologies. Furthermore, the majority of methods of CO_2_ conversion require the use of hydrogen gas, H_2_, which is currently commercially obtained primarily from methane reforming.^8^ The use of H_2_ is associated with high economic and environmental costs as well as substantial safety concerns, in particular flammability and leakage. Furthermore, 8.9 kg of byproduct CO_2_ is produced per kg of H_2_ from methane reforming, resulting in a large carbon footprint.^9^ Therefore, the direct conversion of CO_2_ to value‐added products in the absence of gaseous H_2_ is highly desirable. Perhaps surprisingly, consideration of hypotheses of the origin‐of‐life on Earth may provide a source of inspiration as to how this vision can be realised.

A widely discussed hypothesis is that the origin‐of‐life on Earth occurred around deep‐sea hydrothermal vents, where simple organic molecules can readily form. Temperatures and pressures around these vents can reach 400 °C and 500 bar, and CO_2_ can be present in concentrations up to approximately 500 mmol kg^−1^.^10^, ^11^, ^12^ French first proposed the synthesis of abiotic methane at hydrothermal vents by Fischer–Tropsch‐type catalytic reactions in the presence of iron‐containing minerals.^13^ Generally, hydrocarbons and other organic species in hydrothermal vents form from CO_2_ or other inorganic carbon.^14^ Iron is the most abundant metal in the core of Earth and found throughout the ocean floor,^15^ and its role in the conversion of CO_2_ to organic species has garnered a great deal of interest. It is believed that iron‐bearing minerals and sulphides behave as reductants for CO_2_ to organic species at vent interfaces, with hydrogen provided through water splitting.^16^ Therefore, the activity of bulk iron and iron oxides in this reaction has been widely investigated. This concept has gained further interest in the use of subcritical water to abiotically convert CO_2_ to organic species in laboratory environments.^17^ Hydrothermal reactions take place in sealed pressure vessels in aqueous media at conditions above 100 °C and 1 bar, at which point water exists in a subcritical state.^18^ The properties of subcritical water differ greatly from that of water at room temperature, including high solubility of organic substances and acceleration of acid‐ and base‐catalysed reactions.^19^, ^20^ A decrease in dielectric constant further makes it a good solvent for organic and hydrophobic substances.^19^ The primary focus of CO_2_ reduction in hydrothermal media has been on the synthesis of short‐chained hydrocarbons such as methane, CH_4_ and formic acid, a versatile platform molecule. The reduction of aqueous carbon dioxide by zero‐valent iron at ambient conditions in seawater solution was reported by Hardy and Gillham, who obtained hydrocarbons of chain length up to C_5_ after 500 h.^21^ At shorter reaction times of 40 days, conversion to CH_4_ and short‐chain alkanes in the presence of Fe at a pressure of 1 bar was achieved by Deng et al.^22^ Increased yields of C_1–3_ alkanes in the presence of iron‐containing olivine at 300 °C and 500 bar after 69 days were reported by Berndt et al.^17^ At shorter reaction times of 20 h, Guan et al. synthesised CH_4_ and trace methanol over zero‐valent Fe.^23^ High yields of methanol are desirable because it is an excellent fuel for internal combustion engines and a precursor for the production of formaldehyde, dimethyl ether, acetic acid and other compounds.^24^, ^25^ At mildly hydrothermal conditions up to 200 °C, He et al. found that formic and acetic acids can be obtained with iron powder at reaction times of 2 h.^7^ To illustrate the ability of iron to generate H_2_ gas, Michiels et al. performed a two‐step hydrothermal reaction over zero‐valent Fe powder.^26^ First, H_2_ was produced, after which potassium carbonate, the CO_2_ source, was converted to formate, HCOO, with 78 % conversion and a selectivity in excess of 80 %. Iron oxides have also been found to be effective in this reaction. Chen et al. demonstrated that in the presence of magnetite, Fe_3_O_4_, aromatic compounds are observed at 300 °C,^27^ whereas at higher temperatures of up to 350 °C organic acids are obtained.^28^ In these cases, the role of iron is to promote the reduction of carbon dioxide. However, the reaction is most likely surface‐mediated, progressing through an iron‐stabilised transition state. Therefore, iron can be considered as a catalyst and may also play a role in directing secondary reactions resulting in the formation ≥C_2_ products. It should be noted that other relatively Earth‐abundant metals with low redox potential, such as zinc, are also useful catalysts for CO_2_ reduction.^29^, ^30^ Low‐reduction‐potential metals have been combined with other catalytic substances, particularly nickel^31^, ^32^ and copper,^29^, ^33^, ^34^ to enhance reaction kinetics and yields. Additionally, reducing agents such as glycerol,^35^ biomass derivatives^36^ and microalgae^37^ have been effective in converting CO_2_ to HCOOH.

The studies reported above have predominantly focused on providing support for the hypothesis that reactions around hydrothermal vents contributed to the development of species necessary for the formation of life. However, a detailed understanding of these processes presents the possibility to adapt and optimise these reactions for the synthesis of a wider range of products such as aromatics and longer‐chained cyclic and linear oxygenates from CO_2_. The hydrothermal conversion of CO_2_ in the absence of gas‐phase H_2_ to species such as cyclic oxygenates has not previously been reported. Bulk iron and iron oxides have been widely investigated in origin‐of‐life studies and thus can be used to provide a benchmark for alternative catalysts and reaction conditions. Tailoring the product distribution of this process through judicious selection of catalyst is herein presented for the first time. The use of bifunctional catalysts containing both a metallic and an acidic zeolite component has previously been proposed in other systems as a means of producing longer‐chain hydrocarbons in the gasoline range from gaseous CO_2_ and H_2_ 38 and may therefore also present a route to the formation of larger species in hydrothermal media. In this work, the activity of iron supported on alumina, Fe/Al_2_O_3_, and two zeolite‐supported catalysts, Fe/H‐ZSM‐5 and Fe/HY, as well as the bare zeolites, is investigated. The primary aim is to develop a nature‐inspired, H_2_‐free route to synthesise value‐added organic species from carbon dioxide and water with the aid of iron‐containing catalysts.

## Experimental Section

A range of solid reductants have been utilised for reaction studies. The synthesis and characterisation of these are described in “Solid reductants and catalysts”. The reaction studies and analysis of the reaction products are described in “Reaction testing and product analysis”.

### Solid reductants and catalysts

Two categories of solid reductants/catalysts have been utilised in this work: (i) bulk iron and iron oxides; and (ii) supported iron catalysts. The bulk iron materials were Fe powder (≥99 %, Sigma Aldrich), Fe_2_O_3_ (99 % metal basis, Alfa Aesar) and Fe_3_O_4_ (97 % metal basis, Alfa Aesar); these were used without further treatment or modification. The supported catalysts comprised an alumina supported iron catalyst Fe/Al_2_O_3_ (35 % loading, Johnson Matthey Catalysts) and two zeolite‐supported catalysts (Fe/H‐ZSM‐5 and Fe/HY) synthesised in‐house. The bare zeolites (H‐ZSM‐5 extrudate, SiO_2_/Al_2_O_3_ mole ratio=1:38, ACS material; and H‐zeolite Y powder, SiO_2_/Al_2_O_3_ mole ratio=5.1:1, Alfa Aesar) and a commercial alumina [Puralox SBa200 (Sasol)] were also studied for comparison. The SiO_2_/Al_2_O_3_ mole ratios for the zeolites were supplied by the manufacturers. In the case of the extrudates this ratio refers to the zeolite component; ZSM‐5 was in the H‐form in the supplied extrudates.

#### Catalyst synthesis

Zeolite‐supported iron catalysts were prepared through impregnation employing a method adapted from Lu et al.^39^ Prior to impregnation, H‐ZSM‐5 extrudates were ground with a pestle and mortar. To produce each catalyst, each zeolite (5 g) was mixed with aqueous solution of Fe(NO_3_)_3_⋅9 H_2_O (0.1 m, 100 mL). The mixtures were then stirred for 12 h at 30 °C before being dried overnight at 120 °C. Subsequently, the catalysts were calcined in a furnace (MTI Corporation model KSL‐1200 X) under static air, during which time the temperature was first increased to 120 °C at 10 °C min^−1^ and then held for 1 h, before the temperature was further increased to 550 °C and then held for 5 h. The synthesised catalysts were then cooled to room temperature, again under air.

#### Catalyst characterisation

Brunauer–Emmett–Teller (BET) surface areas of the catalysts were determined by using a 3 Flex Micromeritics Surface Characterization instrument. Prior to analysis, catalysts were dried at 120 °C in a vacuum furnace. Zeolite‐based materials were also exposed to an in situ degasification, during which the temperature was increased to 300 °C at 10 °C min^−1^ and then held for 1 h.

The morphological characteristics of the catalysts, both fresh and post‐reaction, were investigated by SEM on a Jeol JSM‐6010 LA Analytical microscope. The accelerating voltage employed ranged from 15 to 20 kV, and a working distance of 12 mm was used throughout. Catalysts were precoated with gold for 10 s in an Agar Sputter Coater at 0.04 mbar, and a current of 40 mA was applied.

In addition to SEM, the physical structure of the bulk iron and iron oxide catalysts after reaction was also investigated by XRD to identify any changes in the bulk iron composition. XRD patterns were recorded by using a diffractometer (STOE STADI P) operated in transmission mode at a voltage of 20 kV and a current of 5 mA. Data were collected at room temperature in the 2*θ* range from 5 to 39.98° with a step size of 0.020° using MoK_α_ radiation.

Finally, any carbonaceous material (coke) deposited during reaction over the bulk iron materials was evaluated by temperature‐programmed oxidation (TPO) employing a pulse chemisorption system (ChemiSorb 2720, Micromeritics), equipped with a Eurotherm 2416 temperature controller. The TPO method consisted of fluxing in He for 30 min with a flow rate of 25 mL min^−1^ to clean the catalyst surface. Thereafter, the inert gas flow was switched to 5 % O_2_/He. After 20 min, the temperature was increased to 950 °C with a heating rate of 10 °C min^−1^ and held for 30 min.

### Reaction testing and product analysis

In all cases, the reactor (100 mL EZE‐Seal Reactor, Parker Autoclave Engineers®, manufactured from Hastelloy C) was loaded with catalyst (0.56 g) and distilled water (7 mL). GC–MS (Shimadzu QP2010SE, DB1‐MS column, 60 m length, 0.25 mm inner diameter, 0.25 μm film thickness) analysis indicated that the presence of organic matter in the water was negligible prior to reaction. Subsequently, the reactor was twice flushed with CO_2_ (purity 99.99 %, BOC) to eliminate air. The system was then pressurised with CO_2_ to approximately 25 barg (CO_2_/H_2_O mole ratio=0.26) and heated to 300 °C, resulting in an autogenous pressure increase. In studies investigating the influence of CO_2_/H_2_O ratio the reaction temperature was 350 °C, and the step of flushing CO_2_ to eliminate air was omitted. The reaction start‐time was taken as the time at which the impeller (600 rpm) was turned on. After the desired reaction period (4 h) the reaction was quenched by placing a water–ice bath around the reactor.

Analysis of the gas phase was performed at approximately 22 °C by MS (HPR‐20 QIC, Hiden Analytical) (Table S1 in the Supporting Information). The liquid and solid materials were then separated by vacuum filtration. The solid residue was rinsed with water and dried overnight at approximately 110 °C. For the data presented in “Solid reductants and catalysts”, liquid‐phase products were characterised by GC–MS on a Shimadzu QP2012SE. 4‐methyl‐2‐pentanol (98 %, Sigma Aldrich) was used as an internal standard in a ratio of 1 μL to 1.5 mL sample. Quantification was conducted by calibration curves for the species identified. Full experimental details for GC–MS analysis are provided in the Supporting Information.

CO_2_ conversion (*X* ${}_{\mathrm{CO}{}_{2}}$ ) was calculated according to Equation (1) and the selectivity (*S*_i_) according to Equation (2):

(1) $$X{}_{\mathrm{CO}{}_{2}}=\left(n{}_{\mathrm{CO}{}_{2},\mathrm{initial}}-n{}_{\mathrm{CO}{}_{2},\mathrm{final}}/n{}_{\mathrm{CO}{}_{2},\mathrm{initial}}\right)\times 100$$

(2) $$S{}_{i}=\left(n{}_{i}/n{}_{\mathrm{CO}{}_{2},\mathrm{initial}}-n{}_{\mathrm{CO}{}_{2},\mathrm{final}}\right)\times 100$$

in which *n*_i_ is the number of moles of a given product formed; *n* ${}_{\mathrm{CO}{}_{2},\mathrm{initial}}$ is the total number of moles of CO_2_ present initially in both the gas and liquid phase; and *n* ${}_{\mathrm{CO}{}_{2},\mathrm{final}}$ is the total number of moles of CO_2_ after reaction in both the gas and liquid phase.

## Results and Discussion

There are relatively few previous studies on the hydrothermal conversion of CO_2_ in the absence of an additional hydrogen source.^7^, ^17^, ^23^, ^27^, ^28^, ^40^ The majority of these have focussed on investigating the role of such reactions in the production of molecules necessary for the evolution of life on Earth and have hence principally considered the use of bulk iron powder and/or iron oxides as reductants or catalysts. In the present work, the application of such reductants is therefore investigated in detail initially to demonstrate the feasibility of this process in a conventional batch reactor and to provide a benchmark against which to measure future process optimisation around the use of alternative reductants and catalysts. In advance of reaction studies, the following section presents details of the characterisation of the fresh reductants and catalysts.

### Catalyst characterisation

The calculated BET surface areas of the catalysts are shown in Table 1. The surface area of bulk Fe powder is not shown because it was below the measurement limitations of the apparatus. As expected, the bulk iron oxides show substantially lower surface areas than the zeolites and zeolite‐supported catalysts. The deposition of iron on the zeolite surface results in a decrease in surface area, both through a loss of surface sites and through pore blockage.

**Table 1:** Measured BET surface areas of the reductants/catalysts employed. Error is ±2 % of the quoted values.

Catalyst	BET surface area [m^2^ g^−1^]
Fe_3_O_4_	7
Fe_2_O_3_	11
Fe/Al_2_O_3_	74
γ‐Al_2_O_3_	181
Fe/H‐ZSM‐5	354
H‐ZSM‐5	533
Fe/HY	523
H‐Zeolite Y	559

**Figure 1:**
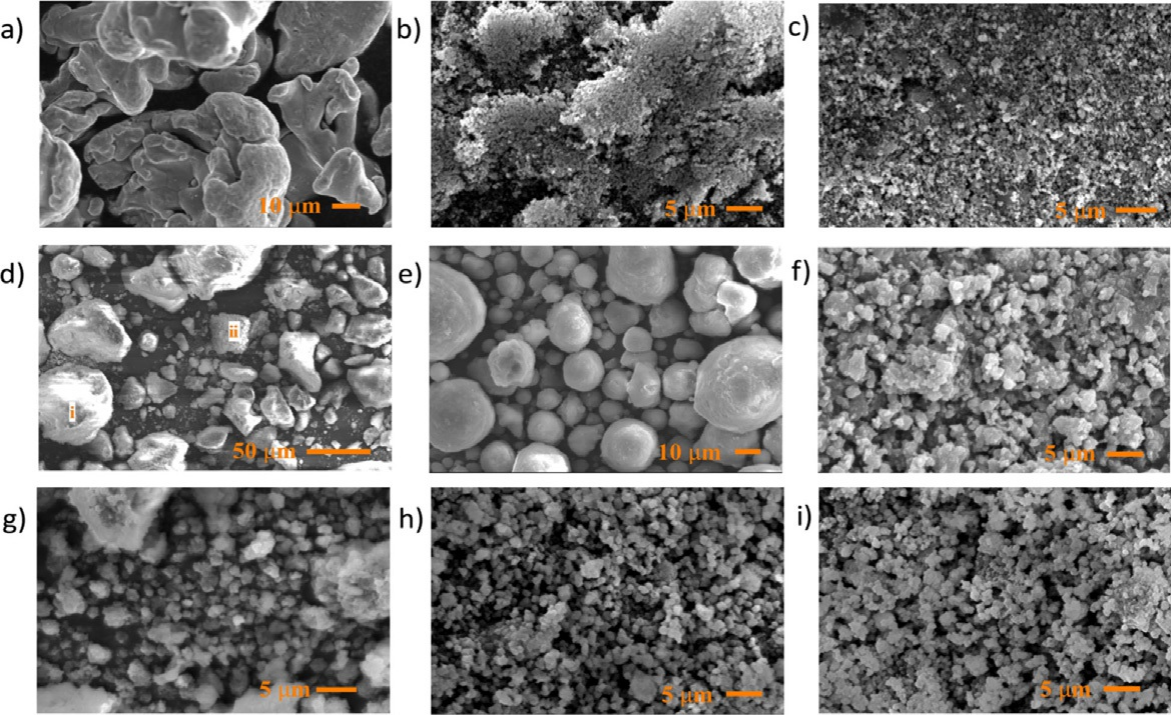
SEM images of the reductants/catalysts employed in the hydrothermal reduction of CO_2_: (a) Fe powder; (b) Fe_3_O_4_; (c) Fe_2_O_3_; (d) Fe/Al_2_O_3_; (e) Al_2_O_3_; (f) H‐ZSM‐5; (g) Fe/ZSM‐5; (h) HY; (i) Fe/HY.

SEM was used to investigate the surface morphology of the catalysts prior to reaction. Representative micrographs are shown in Figure 1. The differing morphology of the various catalysts employed is clearly demonstrated, with iron powder showing both column‐ and plate‐like grains [Figure 1 (a)], iron oxides existing as clusters of smaller particles, Al_2_O_3_ and Fe/Al_2_O_3_ consisting of approximately spherical particles and the zeolite catalysts existing as clusters of varying sizes presenting inter‐particles cavities alongside the intra‐particles channels present within their structure.

### Reactions over bulk iron powder and iron oxides

The hydrothermal conversion of CO_2_ over bulk iron powder and iron oxides provides a benchmark for future studies and a direct correlation to previous work. Herein, particular attention is paid to relative quantities of carbon dioxide and water in the reaction with a view to altering the product distribution, specifically targeting longer‐chained products and aromatic species alongside short‐chained oxygenates such as methanol. Characterisation of the reductants/catalysts after reaction is described in “Evolution of materials during reaction”.

#### Bulk iron powder

**Figure 2:**
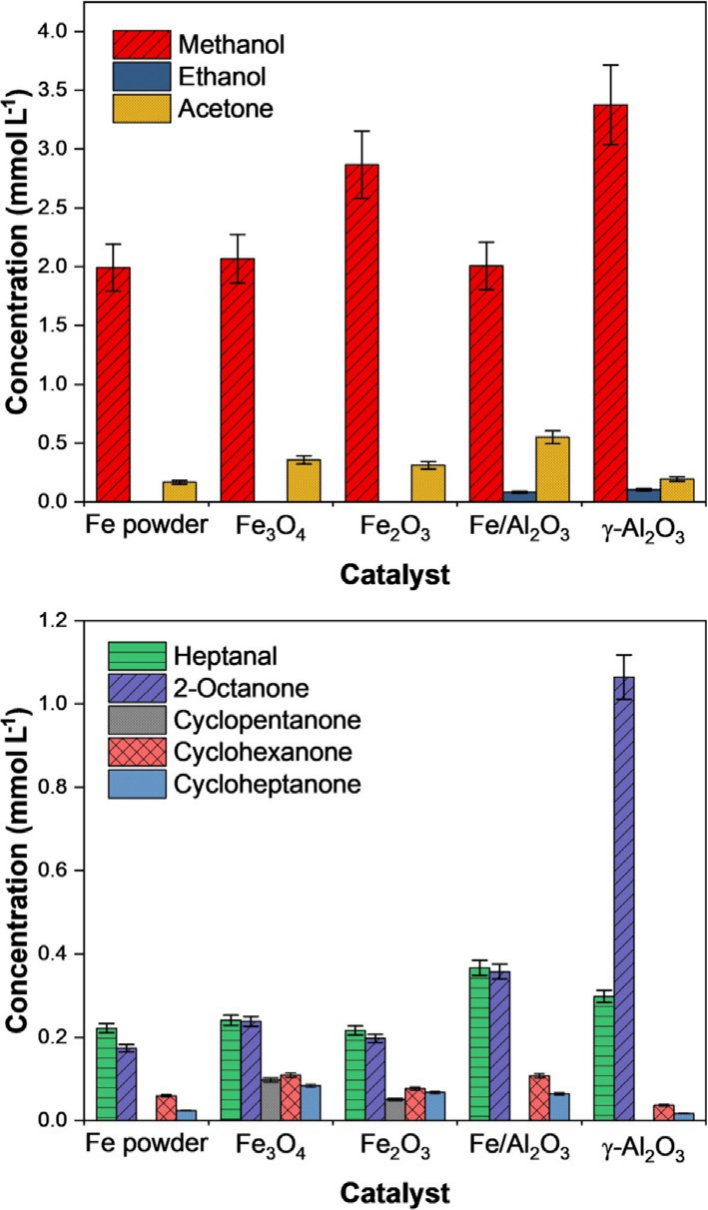
Distribution of liquid‐phase products over iron, iron oxide and alumina‐based reductants/catalysts. Top plot shows low‐carbon‐number products; bottom plot shows higher‐carbon‐number products. Reaction conditions: *T*=300 °C, CO_2_/H_2_O mole ratio=0.26, *t*=4 h, 0.56 g catalyst.

The hydrothermal conversion of CO_2_ over bulk iron powder yielded methanol (2.0 mmol L^−1^) and acetone (0.17 mmol L^−1^), as shown in Figure 2. In addition, under the reaction conditions investigated, longer‐chained species were also produced in comparable amounts; specifically, heptanal (0.22 mmol L^−1^) and 2‐octanone (0.17 mmol L^−1^), alongside trace amounts of cyclohexanone (0.06 mmol L^−1^) and cycloheptanone (0.02 mmol L^−1^). This high selectivity towards methanol is advantageous in the development of processes within the “methanol economy”; however, the observation of significant concentrations of C_7_ and C_8_ products is also of particular interest. These longer‐chained species can act as precursors for the synthesis of a number of products currently derived from fossil resources but are produced here directly from water and carbon dioxide. In addition to liquid‐phase products, the gas‐phase species hydrogen, methane, acetaldehyde, formaldehyde and ethene were identified over bulk Fe powder (Table S1 in the Supporting Information). In total, these represent <1 % of the gas‐phase species in the reactor post‐reaction. In contrast, the quantities of methane and ethene produced were 13 and 18 μmol, respectively. Hydrogen was the most abundant species (2.0 mmol), suggesting that hydrothermal conditions may facilitate water splitting as previously proposed.^16^ An alternative hypothesis is that H_2_ is formed through the water–gas shift (WGS) reaction of CO and H_2_O, which is well‐known to occur over iron‐based materials.^41^, ^42^ In this case, reduction of CO_2_ on the metal surface first leads to the formation of CO and iron oxides, which are active WGS catalysts. Subsequently, in the presence of water, CO can be readily converted to CO_2_ and H_2_ gas. XRD studies in the present work identified that some oxidation of bulk Fe indeed occurs. However, it is noteworthy that H_2_ formation was also observed in reactions over iron‐free catalysts, for example, γ‐Al_2_O_3_ and zeolites (Table S1 in the Supporting Information), which are not known as WGS catalysts. Therefore, at least a portion of H_2_ is proposed to arise from water splitting under hydrothermal conditions.

It is also important to establish if any conversion occurs in the absence of a reductant or catalyst, either induced thermally or promoted by the reactor walls, the latter being an often under‐identified cause of chemical reaction.^43^ The reactor employed herein is constructed from Hastelloy C (58.6 % Ni), and the conversion of CO_2_ into oxygenates and hydrocarbons has previously been demonstrated over nickel.^31^, ^32^ In the absence of a catalyst, the hydrothermal reaction of CO_2_ at 300 °C yielded approximately 1 mmol L^−1^ methanol and approximately 0.07 mmol L^−1^ acetone. Ethanol was also detected in trace amounts; however, higher‐chained products were not observed. Therefore, although some activity is apparent without adding an additional reductant/catalyst, the presence of iron powder substantially increases the quantity of methanol produced and provides a new pathway to the synthesis of longer‐chained products.

The reactivity observed over bulk iron powder therefore confirms the ability of iron to promote the formation of oxygenates from CO_2_ under hydrothermal conditions. This contributes to the body of evidence that such reactions may provide a role in the formation of molecules necessary for the evolution of life on Earth and also provides a valuable baseline from which to compare the behaviour of other reductants and catalysts in the ongoing drive to develop sustainable industrial routes to hydrocarbons and oxygenates taking inspiration from such natural reactions.

#### Bulk iron oxides

The active surface phase and oxidation state of iron under hydrothermal reaction conditions is the subject of debate with, for example, the formation iron oxyhydroxide having been previously characterised in both natural environments around hydrothermal vents and in laboratory studies under aqueous conditions at elevated temperature.^44^, ^45^, ^46^, ^47^ Because iron has been proposed to act directly as a reductant and oxygen sink in this reaction it might be expected that more oxidised forms of iron are less favourable in promoting CO_2_ hydrogenation and polymerisation. Fe^II/III^ and Fe^III^ oxides (Fe_3_O_4_ and Fe_2_O_3_, respectively) have therefore been investigated in place of bulk iron powder.

As bulk materials with no, or low, porosity, Fe_3_O_4_ and Fe_2_O_3_ have intrinsically low surface areas of 7 and 11 m^2^ g^−1^ respectively (Table 1). As with Fe powder, however, they demonstrate clear and measurable activity towards CO_2_ conversion. The CO_2_ conversions achieved over Fe_3_O_4_ and Fe_2_O_3_ were 14 and 11 % respectively, compared with 7 % over bulk Fe. The higher conversion over Fe_3_O_4_ is achieved despite having a lower surface area than Fe_2_O_3_, whereas conversion over Fe powder is 64 % of that achieved over Fe_2_O_3_ although the former has a surface area below the measurable limit. It is therefore apparent that conversion does not exhibit a simple direct correlation with surface area, and that among the oxide materials the more reduced species has higher activity, as predicted. However, all species are active for the hydrothermal conversion of CO_2_.

Figure 2 shows the product distribution observed in the liquid phase over the bulk oxides. The major product formed in both cases was methanol with a concentration of approximately 2–3 mmol L^−1^, similar to that over bulk iron powder, with Fe_2_O_3_ yielding the highest concentration. Acetone production was also observed. The methanol/acetone ratios over Fe, Fe_3_O_4_ and Fe_2_O_3_ are 12:1, 6:1 and 9:1, respectively. The production of the C_3_ ketone is therefore more favourable, with respect to methanol, over the oxides. This trend follows the increase in CO_2_ conversion over the three materials; therefore, acetone may be formed through successive CO_2_ addition to methanol.

Analysis of the reactor headspace after reaction indicated the presence of methane, acetaldehyde, formaldehyde and ethene, alongside CO (Table S1 in the Supporting Information). These products represent 12 and 9 % of the gas‐phase species over Fe_3_O_4_‐ and Fe_2_O_3_‐promoted reactions, respectively. This compares to <1 % over bulk Fe; this difference is a consequence predominately of elevated of CO formation over the oxides for which the selectivity to CO was 79 and 74 %, respectively. Fe_3_O_4_ and Fe_2_O_3_ also exhibited higher hydrocarbon production, yielding approximately 18 μmol CH_4_ and in excess of 30 μmol C_2_H_4_ (Table S1 in the Supporting Information). The formation of CO from CO_2_ over iron materials is well known, in particular through the reverse water–gas shift reaction (RWGS). Indeed, RWGS has been proposed as key step in the synthesis of higher hydrocarbons from CO_2_ through a modified Fischer–Tropsch reaction [Eq. (3)] involving stepwise hydrogenation of CO_2_ and CO with gas‐phase H_2_ over supported iron catalysts:^48^, ^49^, ^50^, ^51^

(3) $$1.\;\;\mathrm{CO}{}_{2}+H{}_{2}\vec{\leftarrow }\mathrm{CO}+H{}_{2}O\;\;\;\Delta H{}_{573K}=38\;\mathrm{kJ}\;\mathrm{mol}{}^{-1}2.\;\;\mathrm{CO}+2\;H{}_{2}\to \left(\mathrm{CH}{}_{2}\right){}_{n}+H{}_{2}O\;\;\;\Delta H{}_{573K}=-166\;\mathrm{kJ}\;\mathrm{mol}{}^{-1}$$

Methanol, ethanol and longer‐chained alcohols and other oxygenates can also be produced through this route where step 2 in Equation (3) becomes Equation (4):

(4) $$2\;\mathrm{CO}+4\;H{}_{2}\to \mathrm{CH}{}_{3}\mathrm{CH}{}_{2}\mathrm{OH}+H{}_{2}O$$

with >C_2_ alcohols formed through polymerisation of ethanol.^52^, ^53^, ^54^, ^55^ The formation of CO through the RWGS reaction may therefore provide an alternative route to the formation of C_2+_ oxygenates under hydrothermal conditions. In analogous gas‐phase reactions over solid catalysts it is known that the synthesis of C_2+_ species from CO_2_/H_2_ mixtures first involves in situ formation of CO as a key intermediate through the RWGS reaction.^56^ However, in the present work the relative absence of CO over bulk Fe powder suggests that it may not be a prerequisite and that higher products may be formed directly from CO_2_. To confirm the mechanistic role of CO in long‐chain oxygenate formation, future studies should consider spiking the reaction with increasing amounts of CO.

#### Synthesis of aromatics over bulk iron

**Figure 3:**
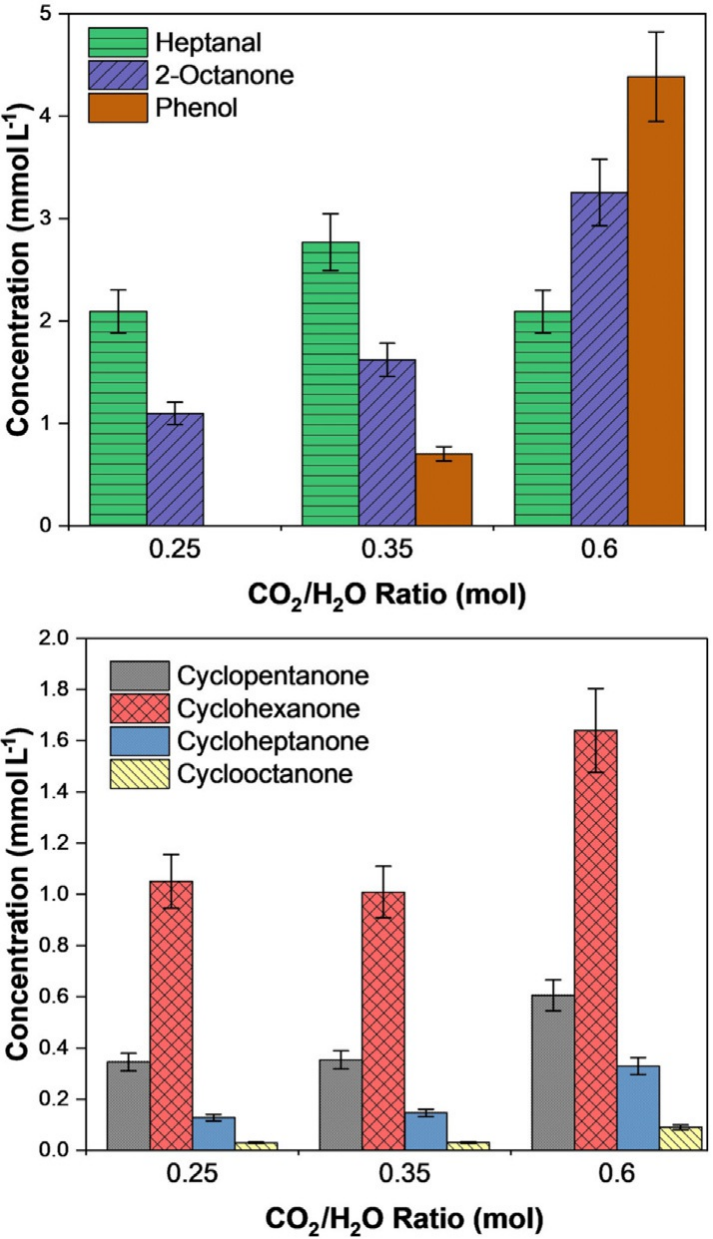
Distribution of liquid‐phase products over iron powder with varying CO_2_/H_2_O ratios. Top plot shows linear and aromatic products; bottom plot shows (non‐aromatic) cyclic products. Reaction conditions: *T*=350 °C, *t*=4 h, 0.56 g catalyst.

Aromatic compounds have higher C/H ratios than linear or other cyclic species, and therefore it may be expected that a higher reaction temperature and increasing the CO_2_/H_2_O ratio in the reactor could direct the reaction towards the formation of such products. The synthesis of phenol under hydrothermal conditions has previously been observed by Tian et al., with yields of up to 0.8 mol % (0.4 mmol L^−1^) observed with sodium bicarbonate as the CO_2_ source and 1.2 mol % (1.1 mmol L^−1^) with gas‐phase CO_2_ directly.^57^, ^58^ Figure 3 compares the production of phenol to other high‐carbon‐number (C_5–8_) species, both linear and cyclic, in the present work over Fe powder. It is clear from these data that upon increasing CO_2_/H_2_O from 0.25 to 0.35 and then further to 0.60 the production of phenol is significantly enhanced, with a yield of 4.5 mmol L^−1^ at the highest ratio; this is significantly higher than previously observed yields. At the same time, the reaction increasingly favours longer‐chained species at higher CO_2_/H_2_O ratios with a decrease in the production of heptanal coupled with an increase in the production of 2‐octanone. The mole ratio of 2‐octanone to heptanal increases from 0.5:1 at a CO_2_/H_2_O ratio of 2.5 to 1.6:1 at a CO_2_/H_2_O ratio of 0.6.

These data strongly support the hypothesis, tested here for the first time, that the product distribution can be tailored to produce not only longer hydrocarbon chains but also desirable aromatic species, which are particularly valuable given the current reliance on crude oil for their production and the current challenges in producing aromatic species from simple molecules such as CO_2_ by conventional routes.

#### Evolution of materials during reaction

**Figure 4:**
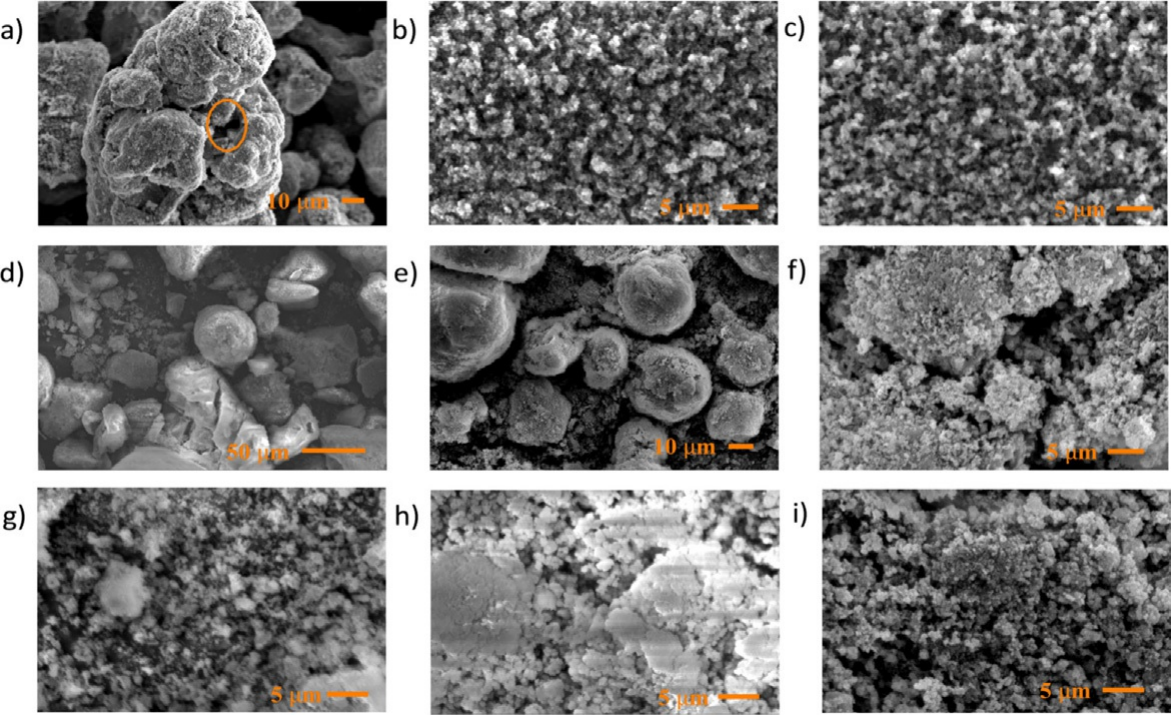
Post‐reaction SEM images of the reductants/catalysts employed in the hydrothermal reduction of CO_2_: (a) Fe powder; (b) Fe_3_O_4_; (c) Fe_2_O_3_; (d) Fe/Al_2_O_3_; (e) Al_2_O_3_; (f) H‐ZSM‐5; (g) Fe/ZSM‐5; (h) HY; (i) Fe/HY. The circled area in (a) highlights an area identified as containing carbonaceous deposits.

SEM was used to determine if any significant morphological changes were induced in the catalysts by the processing conditions employed [Figure 4 (a–c)]. Comparing Figures 1 and 4, there are only very limited morphological changes evident after reaction. Most notable is the increase in surface roughness, in part ascribable to the presence of surface carbonaceous deposits (see below).

**Figure 5:**
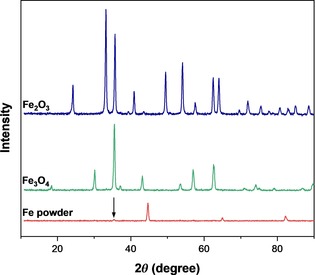
XRD patterns for spent Fe powder, Fe_3_O_4_ and Fe_2_O_3_ catalysts. The arrow indicates a low‐intensity peak at 35.36° indicative of Fe_3_O_4_.

XRD was employed to investigate changes in the iron phases present in the bulk iron and iron oxides after reaction. As shown in Figure 5, the peaks observed in the post‐reaction Fe_3_O_4_ and Fe_2_O_3_ XRD patterns correspond to the original Fe_3_O_4_ and Fe_2_O_3_ crystalline phases, respectively. Spent Fe powder exhibited the three characteristic peaks of metallic α‐Fe at 2*θ*=44.68, 65.03 and 82.21°. In addition, a low‐intensity peak was observed at 35.36° (indicated by an arrow in Figure 5), which can be assigned to Fe_3_O_4_.^59^ Thus, these findings suggest that a slight oxidation of Fe powder catalyst may have occurred during the hydrothermal reaction. Although no significant changes were observed in the bulk phase of the oxide catalysts, and therefore they do not undergo significant bulk oxidation, transformation in the composition of the catalyst surface, such as changes in the oxidation state of the Fe, may have occurred.

**Figure 6:**
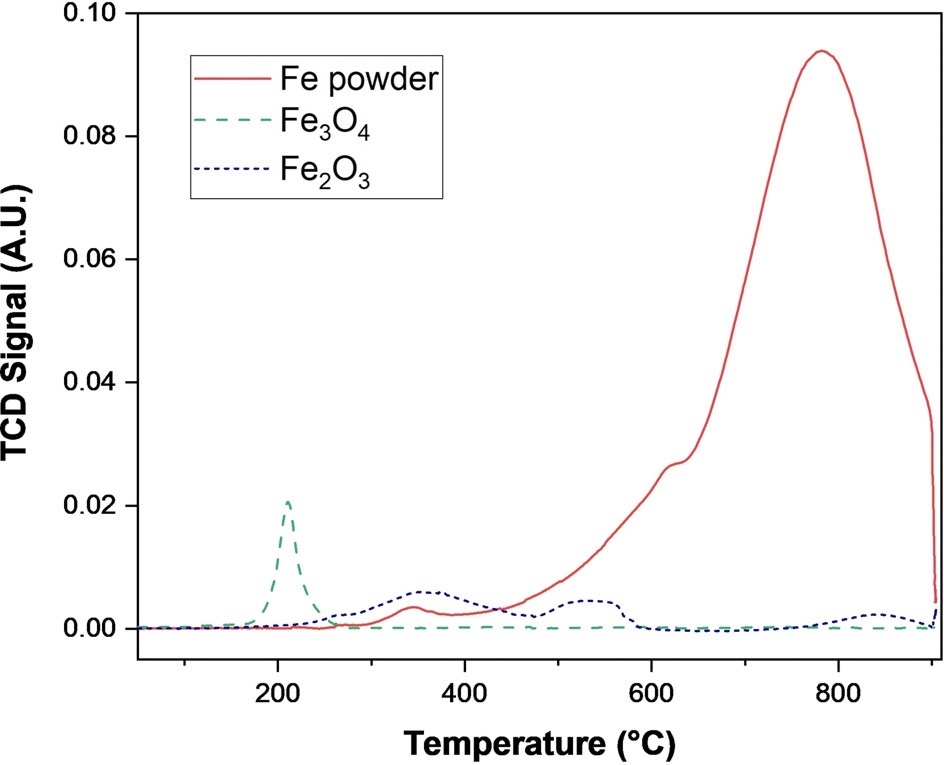
TPO profile of spent Fe powder and Fe oxide catalysts. Reaction conditions: *T*=300 °C, CO_2_/H_2_O mole ratio=0.26, *t*=4 h, 0.56 g catalyst. Mass of spent catalyst employed in TPO analysis=35–45 mg.

The nature and quantity of carbon deposited on the catalysts was investigated through TPO. TPO profiles for iron powder, Fe_3_O_4_ and Fe_2_O_3_ after 4 h of reaction are shown in Figure 6. There are clear differences in both the total amount of carbon deposited and the oxidation temperature—and hence structure—of the coke. Iron powder, despite having the lowest surface area, has both the greatest quantity of, and most structured, coke deposits. The latter is revealed by the high oxidation temperature, with the major peak centred at approximately 750 °C, alongside a shoulder at approximately 600 °C. This temperature is typical of graphitic structures.^60^ These data are also consistent with the carbon balance over the catalysts, which shows an imbalance between the quantity of carbon introduced as CO_2_ initially and that present in the liquid and gas phase after reaction. The difference between the initial and final carbon quantities was 6.5 % over Fe and 2.1 and 2.0 % over Fe_3_O_4_ and Fe_2_O_3_, respectively. This greater imbalance over Fe is in line with the TPO data showing more retained carbon on the Fe surface as compared to the oxides.

Comparing the two bulk oxides, the TPO profile of Fe_3_O_4_ exhibits a single symmetric peak at approximately 260 °C, typical of amorphous carbon deposits.^60^ In contrast, the Fe_2_O_3_ profile exhibits three broad peaks at 350, 550 and 850 °C corresponding to different surface carbon species. The lower‐temperature peaks are significantly larger in area than that at 850 °C, indicating that overall the carbon deposits formed over Fe_2_O_3_ are dominated by less structured materials than those formed over iron powder.

#### Summary of studies over bulk iron/iron oxides

Bulk iron was confirmed as possessing the ability to promote the hydrothermal conversion of CO_2_ in the absence of gas‐phase H_2_, producing methanol as the major product. Additionally, the product distribution can be tailored by varying reaction conditions, with enhanced CO_2_/H_2_O ratios yielding the valuable aromatic product phenol.

Despite being in a higher oxidation state than bulk iron and hence having reduced capacity as a reductant and oxygen sink, it is also apparent that Fe_2_O_3_ and Fe_3_O_4_ have activity towards CO_2_ conversion under hydrothermal conditions. Comparing the oxides, the more oxidised Fe_2_O_3_ does show the lowest conversion, which may in part be related to its higher oxidation state. The surface‐mediated reaction is, however, clearly dependent on a number of underpinning factors, and a future detailed study, ideally employing advanced operando methods, would be necessary to deconvolute these influences. That more oxidised forms of iron are not inactive is a critical observation and shows that the system will not be deactivated directly through the surface oxidation of iron in high‐temperature water.

### Reactions over supported catalysts

Although iron and iron compounds such as iron oxides are present naturally around deep sea hydrothermal vents, judicious selection and design of catalysts presents an opportunity to improve the efficiency of this process and to target a broader range of products. To exploit this reactivity as a means of synthesising alcohols and other oxygenate products from CO_2_, it is necessary to optimise the catalyst and reductants used. Industrial heterogeneous catalysts are typically prepared with the active species dispersed onto a support, resulting in greater availability of the active metal surface for reaction. Herein, an alumina‐supported iron catalyst is tested to compare to the bulk oxides, and zeolite‐supported iron catalysts are investigated as potential bifunctional materials to alter the product distribution. The use of bifunctional catalysts containing both an acidic zeolite component has previously been proposed as a means of upgrading the products from Fischer–Tropsch synthesis from CO_2_ to longer‐chain hydrocarbons in the gasoline range.^38^ This employs a sodium‐promoted iron catalyst for the initial CO_2_ conversion through RWGS and Fischer–Tropsch reactions, whereas the zeolite component catalyses oligomerisation, isomerisation and aromatisation reactions. Therefore, zeolite‐supported catalysts may produce longer‐chained or cyclic species. Bare alumina and the zeolite supports have also been tested for comparison.

#### Alumina and alumina‐supported iron

Figure 2 shows the extent of production of both short‐ and long‐chained oxygenate products over Fe/Al_2_O_3_ and Al_2_O_3_ alongside the bulk iron and iron oxides. Fe/Al_2_O_3_ shows similar yields of products to the bulk materials overall, but with somewhat enhanced production of longer‐chained products. The methanol/acetone ratio is 3.7:1, lower than that over the bulk materials, whereas greater quantities of heptanal and 2‐octanone are also observed. This is achieved despite a CO_2_ conversion of only 1.1 %. This trend is even more pronounced over γ‐Al_2_O_3_, which yields 1.1 mol L^−1^ 2‐octanone; over six times that produced in the presence of iron powder despite a CO_2_ conversion of only 7.7 %. Acid sites on γ‐Al_2_O_3_ therefore favour the formation of long‐chained products, and the presence of iron active sites is not a prerequisite. CO_2_, H_2_, H_2_O, acetaldehyde and formaldehyde are all observed as products in the gas phase (Table S1 in the Supporting Information). As with the bulk materials, SEM data [Figure 4 (d, e)] show limited differences between the pre‐ and post‐reaction samples, with some agglomeration observed to have taken place for Fe/Al_2_O_3_.

#### Zeolite and zeolite‐supported iron

**Figure 7:**
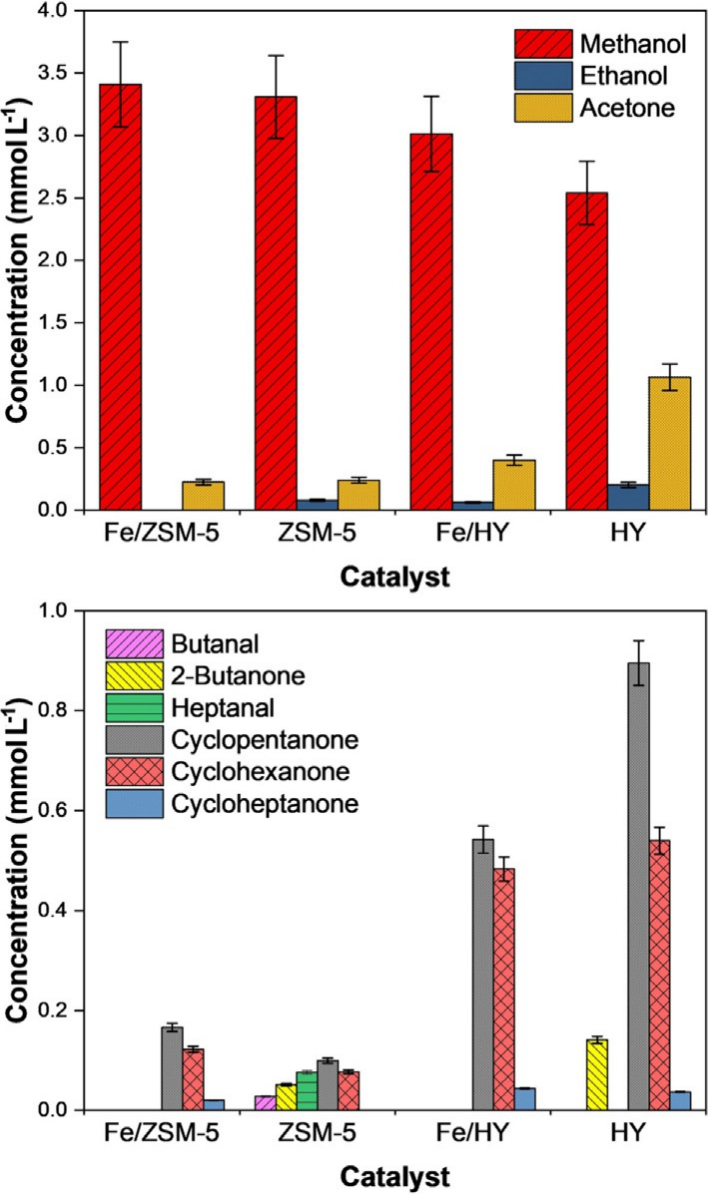
Distribution of liquid‐phase products over zeolite and zeolite‐supported iron catalysts. Top plot shows low‐carbon‐number products; bottom plot shows higher‐carbon‐number products. Reaction conditions: *T*=300 °C, CO_2_/H_2_O mole ratio=0.26, *t*=4 h, 0.56 g catalyst.

The use of zeolite‐supported iron catalysts allows the benefits of a bifunctional catalyst to be probed, in which the zeolite acid sites may catalyse secondary reactions of the primary alcohol products. Figure 7 quantitatively compares the amount of both short‐ and long‐chained oxygenates produced over the zeolite and iron–zeolite catalysts. As with bulk iron powder and iron oxides, methanol is the organic product produced in the greatest quantity, with all four zeolite‐based materials yielding approximately 3 mmol L^−1^. The production of acetone shows a clear dependence on the nature of the zeolite used, and hence on zeolite acidity. Both Fe/H‐ZSM‐5 and H‐ZSM‐5 produced <0.25 mmol L^−1^ whereas over the less acidic Fe/HY and HY, >0.5 mmol L^−1^ and >1 mmol L^−1^ were produced, respectively. Similar gas‐phase species were evolved as over iron and alumina materials (Table S1 in the Supporting Information), and SEM data again show only limited changes after reaction such as the presence of some carbonaceous deposits.

Comparing the production of longer‐chained products over zeolite‐based catalysts to that over the bulk iron powder and bulk iron oxides (Figure 2), a clear difference is the presence of cyclic ketones with five to seven carbon atoms in significant concentrations; for example, eighteen times more cyclopentanol was produced over HY than over Fe_2_O_3_. In contrast, linear species in the same size range were present in lower concentrations; for example, H‐ZSM‐5 yielded 0.07 mmol L^−1^ of heptanal. It is also notable that the unmodified zeolites have comparable performance to the iron‐impregnated zeolites, and indeed unmodified HY exhibits the greatest yields of cyclopentanone and cyclohexanone among all materials investigated. This supports the conclusion of studies on γ‐Al_2_O_3_ (see above) that iron is not required for the hydrothermal conversion of CO_2_ and that active sites on zeolites and alumina are also capable of effecting this transformation.

The formation of cyclic compounds most likely occurs through cyclisation reactions of linear species and can hence be considered as a secondary process. Zeolites are well‐known cyclisation catalysts; for example, cyclisation is a key step in the methanol‐to‐hydrocarbons reaction over H‐ZSM‐5,^61^, ^62^ the mechanism of which is widely discussed. The cyclisation of surface‐adsorbed carbonaceous species has also been identified as a key step in the formation of coke deposits on zeolites.^63^ The tendency to form larger‐carbon‐number rings in HY compared with H‐ZSM‐5 may be ascribed to the larger pore size of the former. Detailed theoretical studies of hydrocarbon oligomerisation–cyclisation reactions over zeolite catalysts have previously provided insights into the reaction mechanism,^64^, ^65^, ^66^ with hydrocarbons proposed to adsorb at framework oxygen species in the zeolite structure. Subsequent dehydrogenation of the cyclic products through a hydride transfer mechanism can yield aromatic products such as those observed in this work under alternative reaction conditions. This latter reaction can be enhanced in the presence of CO_2_, which can act as a hydrogen acceptor.^67^ The product concentrations in the liquid phase after reaction compare favourably with alternative liquid‐phase processes. The electrochemical conversion of CO_2_ to methanol provides an alternative route to this key oxygenate intermediate. Reported methanol concentrations achieved electrochemically range from 0.001 to 6.5 mmol L^−1^.^25^ The results herein, albeit under very different experimental conditions, produce a final liquid‐phase product that compares well with these despite being at an early proof‐of‐concept stage. This highlights the potential role for hydrothermal CO_2_ conversion in supporting the methanol economy, in addition to its ability to yield higher hydrocarbons and aromatics.

#### Summary of studies over supported catalysts

Alumina and zeolites are both observed to enhance the production of longer‐chained and cyclic species, respectively. This suggests that acid sites present on both of these materials play a key role, for example, through catalysing oligomerisation reactions, and that metallic sites are not a prerequisite for catalytic activity. Supporting the conclusion that acid sites play a critical role, complementary studies on carbon‐based catalysts lacking acidic functionalities do not show the production of longer‐chained species.^68^ Notably, zeolite‐based catalysts promoted the formation of cyclic ketones. To the best of our knowledge this represents the first report of such products arising from hydrothermal CO_2_ conversion. These results therefore confirm the possibility to alter, and hence tailor, product distribution through the development of robust structure–performance relationships. To this end, the following section presents initial hypotheses as to the reaction mechanism.

### Reaction mechanism

The observation of longer‐chained products (up to C_8_) in the studies reported herein is highly significant. The production of aromatic and cyclic species is also of great value; however, these most likely form through secondary reactions of linear species, for instance, cyclisation over acid sites present on zeolite supports. It is therefore desirable to understand the reaction mechanism leading to the formation of these longer‐chained species on iron surfaces to ultimately develop structure–performance relationships and hence inform future catalyst design.

**Scheme 1:**
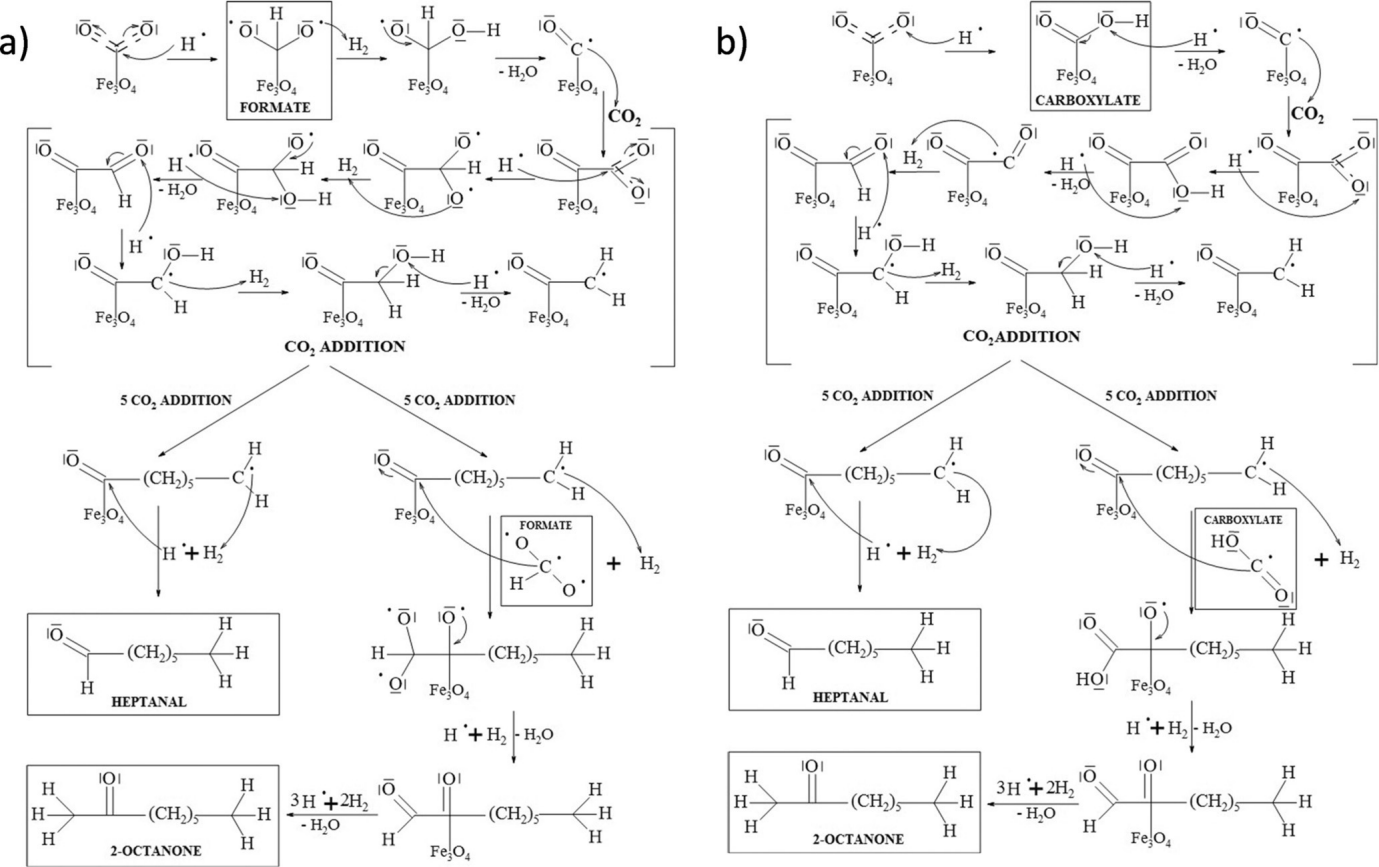
Reaction mechanism for the production of heptanal and 2‐octanone via (a) a formate intermediate and (b) a carboxylate intermediate.

It is possible that the reaction proceeds via either formate (HCOO) [Scheme 1 (a)] or carboxylate (COOH) as an intermediate [Scheme 1 (b)]. It has previously been reported that the adsorption of HCOO on iron surfaces is more stable than that of carboxylate.^69^, ^70^ Thus, it may be expected that the first step in the mechanism of formation of heptanal and 2‐octanone is the formation of formate. However, it has been observed (e.g., Figure 2) that liquid species formed during the hydrothermal conversion of CO_2_ in the present work maintained the C=O double bond. This is consistent with the carboxylate mechanism for which the C=O bond is preserved through all steps.

**Scheme 2:**
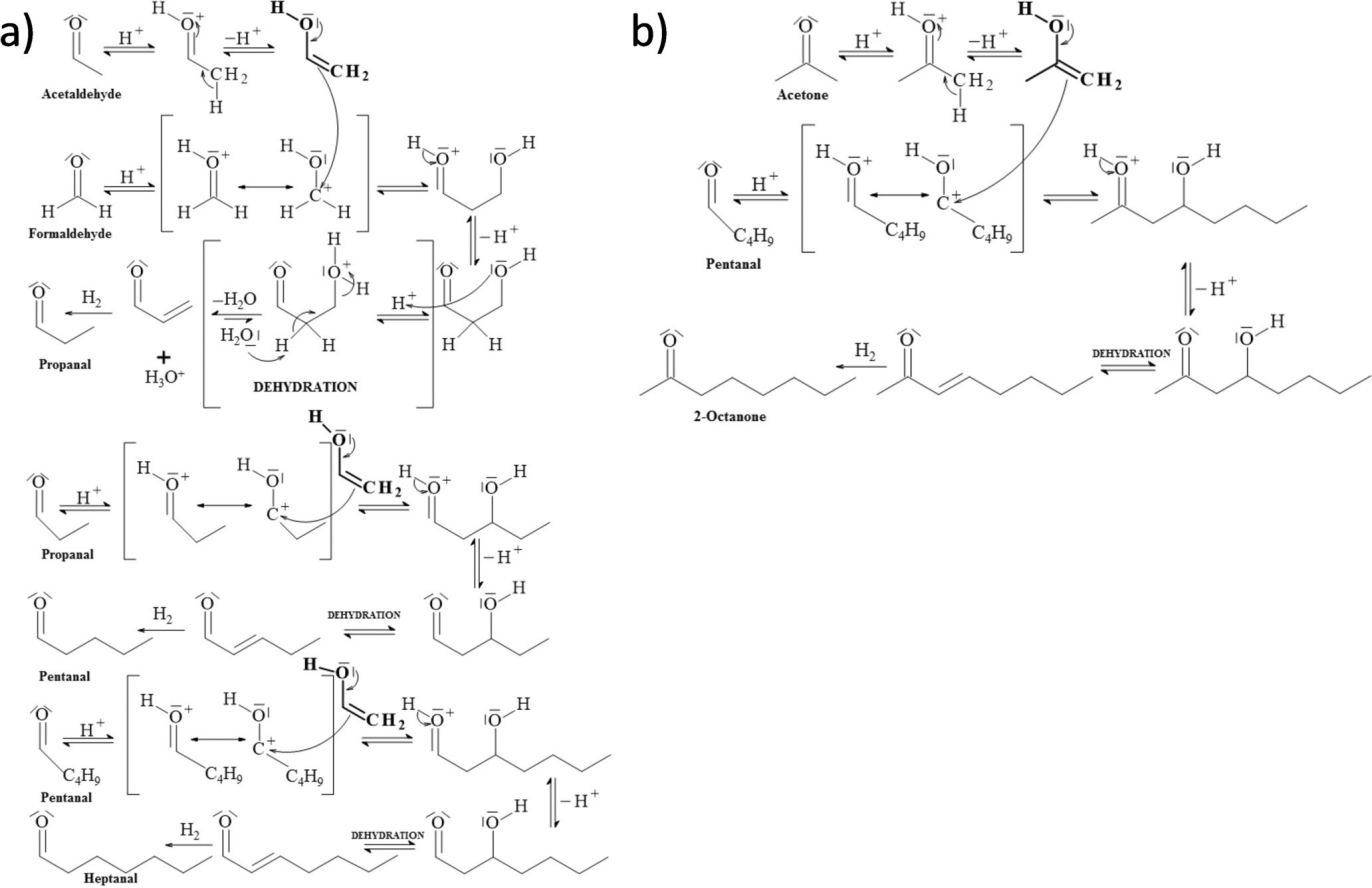
Reaction mechanism for the production of (a) heptanal and (b) 2‐octanone through aldol condensation reactions.

A third possibility that could explain the observation of heptanal and 2‐octanone is that the mechanism may proceed through aldol condensation reactions [Scheme 2 (a) and (b), respectively]. The initial reactants for this process are short‐chained oxygenates such as acetaldehyde, formaldehyde and acetone, all of which are identified as products in the present work. The identification of these crucial intermediates in the reaction from CO_2_ to longer‐chained oxygenates provides some support for this hypothesis. It is, however, notable that branched‐chained species are common products of aldol condensations and that in the present work such branched species were not observed. In the microporous zeolite catalysts this may be ascribed to product shape‐selectivity;^71^ this, however, is unlikely to be applicable to, for example, bulk Fe powder for which heptanal and 2‐octanone are also observed.

To provide further insights into the reaction mechanism the influence of adding oxygenates (methanol and 2‐propanol) to the original reaction mixture and also of replacing water entirely with methanol and 2‐propanol has been investigated. These data show that the presence of alcohols enhances the production of long‐chained species such as heptanal and 2‐octanone (Figure S1 in the Supporting Information) and also results in the formation of additional long‐chained species (Figure S2 in the Supporting Information). However, at least a significant fraction of this conversion can be ascribed to the direct reaction of the alcohols rather than their reaction with CO_2_, as shown by studies under a He atmosphere (Figure S3 in the Supporting Information). The conversion of methanol to higher oxygenates may occur through the condensation of intermediates such as formaldehyde and formyl rather than through the decomposition of methanol into CO and H_2_ followed by a higher‐alcohol synthesis (HAS) route. Therefore, in this case, CO_2_ may promote the formation of the reaction intermediates, and thus, through subsequent condensation, enhance the production of higher oxygenates. The influence of other oxygenates such as sugars, alcohols, aldehydes and acids on the reduction of CO_2_ (from NaHCO_3_) has also previously been investigated by other researchers targeting the production of formic acid.^36^ In those studies, the oxygenates were concluded to act as a hydrogen donor for CO_2_ reduction. The data presented herein therefore indicate that a number of competing parallel reaction mechanisms may operate. It is not yet clear which of these mechanisms dominates. This is a key area for future investigations and will facilitate the development of new reductants and catalysts to optimise this process.

## Conclusions

This work has detailed proof‐of‐concept studies that have confirmed the ability of iron and iron‐based materials to promote the hydrothermal conversion of carbon dioxide to oxygenate species in the absence of gas‐phase hydrogen. Direct inspiration for this work is drawn from reactions that take place in the natural environment around hydrothermal vents. Crucially, the ability to direct the product distribution of this reaction through the choice of catalysts and reaction conditions has been demonstrated for the first time, with significant scope for future process optimisation. For instance, nickel and cobalt are also known to activate CO_2_, and the acidity and/or pore size of support materials can direct for specific carbon‐chain length or ring size. A feature of this process is the ability to target either methanol production, which underpins the so‐called “methanol economy” or longer‐chained species including aromatics (phenol) and cyclic ketones, the production of which by this route had not previously been reported.

## Conflict of interest

*The authors declare no conflict of interest*.

## Supporting information

As a service to our authors and readers, this journal provides supporting information supplied by the authors. Such materials are peer reviewed and may be re‐organized for online delivery, but are not copy‐edited or typeset. Technical support issues arising from supporting information (other than missing files) should be addressed to the authors.

Supplementary
