# Supplementary material for: H2‐free Synthesis of Aromatic, Cyclic and Linear Oxygenates from CO2
Source: ChemSusChem. 2020 Jan 7;13(3):647–58. doi: 10.1002/cssc.201902340 (PMC7027563; doi:10.1002/cssc.201902340)
Supplement: Supplementary file 1 — Supplementary [file CSSC-13-647-s001.pdf]

CHEMISTRY & SUSTAINABILITY
CHEMSUSCHEM
ENERGY & MATERIALS

Supporting Information

H_2-free Synthesis of Aromatic, Cyclic and Linear Oxygenates from CO_2

Laura Quintana Gomez,[a, b] Amal K. Shehab,[a] Ali Al-Shathr,[a] William Ingram,[a] Mariia Konstantinova,[a] Denis Cumming,[a] and James McGregor*[a]

cssc_201902340_sm_miscellaneous_information.pdf

Mass Spectrometry Data

Table S1. Indicative quantities of species detected by mass spectrometry in the gas-phase following the hydrothermal conversion of CO_2 in sub-critical water. Reaction conditions: T = 300 °C, CO_2:H_2O mole ratio = 0.26, t = 4 h, 0.56 g catalyst. 'n.d.' indicates that a substance was not detected.

Catalyst	Gas phase composition (mmol)							
	H_2	H_2O	CO_2	CO	CH_4	C_2H_4O (acetaldehyde)	CH_2O (formaldehyde)	CH_2O_2 (formic acid)	C_2H_4 (ethene)
**Fe powder**	2.0	4.4	90.9	n.d.	<0.1	0.2	0.1	<0.1	<0.1
**Fe_3O_4**	0.2	1.4	83.8	11.1	<0.1	0.3	<0.1	n.d.	<0.1
**Fe_2O_3**	0.3	1.5	86.2	8.2	<0.1	0.3	<0.1	n.d.	<0.1
**Fe/Al_2O_3**	0.1	1.1	95.0	0.3	<0.1	0.4	0.1	n.d.	<0.1
**γ-Al_2O_3**	0.3	0.8	99.0	n.d.	n.d.	0.4	0.1	n.d.	n.d.
**Fe/H-ZSM-5**	0.2	4.4	97.1	n.d.	<0.1	0.3	0.1	<0.1	<0.1
**H-ZSM-5**	0.1	0.3	100.0	n.d.	<0.1	<0.1	<0.1	<0.1	<0.1
**Fe/HY**	0.1	0.3	99.6	n.d.	n.d.	<0.1	<0.1	n.d.	n.d.
**H-zeolite Y**	0.2	0.3	99.5	n.d.	n.d.	0.3	<0.1	n.d.	n.d.

GCMS Analysis

In all GCMS analysis of liquid products, He was used as a carrier gas with a linear velocity of 30 cm s^{-1}; The temperature of the injection port was fixed at 250 °C and the oven temperature programme consisted of several steps, *vide infra*.

For experiments investigating the influence of different catalysts (Section 3) three different temperature programmes were used to analyse shorter and longer-chained products. For short-chained products (up to C_3 but excluding ethanol), a split ratio of 300 was used. The oven programme consisted of holding the temperature at 40 °C for 1 min and then ramping at 10 °C min^{-1} to 63 °C and before holding for a further minute. Thereafter the temperature was increased initially to 100 °C at 10 °C min^{-1} and finally to 300 °C at 15 °C min^{-1}. To analyse ethanol a split ratio of 20 was employed with the following temperature programme. The column oven temperature was initially held at 40 °C for 1 min. With a ramp of 5 °C min^{-1} the temperature was increased first to 63 °C and held at this temperature for 1.00 minute and then increased to 100 °C. Finally, the temperature was raised to 220 °C at a rate of 15 °C min^{-1}. Longer chained products were analysed using a split ratio of 50 while the initial oven temperature was set to 40 °C. Then the temperature was increased successively to 87, 91 and 103 °C at a rate of 10 °C min^{-1} and held at these temperatures for 1.00, 1.50 and 1.00 minutes respectively. Then, the temperature was increased to 150 °C at 10 °C min^{-1} and 300 °C at 15 °C min^{-1}. The oven was maintained at 300 °C for a further 1.00 min.

For experiments with varying CO_2:H_2O ratios (Section 3.2.3), liquid samples were injected in the GCMS with a split ratio of 30. For the temperature programme, the temperature was initially held at 50 °C for 1 min. Thereafter, the temperature was increased at a rate of 15 °C min^{-1} to 78, 89 and 100 °C, holding these temperatures for 2.50, 1.00 and 2.00 min respectively. Finally, the temperature was increased to 150 °C at 10 °C min^{-1} and held constant for 1.00 min.

Influence of Oxygenates on the Hydrothermal Conversion of CO_2

In order to investigate the influence of short-chained oxygenates on the reaction mechanism methanol (≥ 99.8 %, Sigma Aldrich) and isopropaonol (≥ 99.8 %, Sigma Aldrich) were added alongside water in the starting reaction mixture, using 0.56 g Fe powder as a catalyst, a reaction temperature of 300 °C and an initial CO_2 pressure of 25 barg. 30 mol% in water. Ratios of 30 mol% methanol in water and 10 mol% of isopropanol in water were investigated. These mole percentages were selected based on the highest degree of structuring of hydrogen-bonded alcohol/water networks calculated by Li *et al.* (*J. Phys. Chem. B* 2014, **118**, 34, 10156-10166). For comparison the pure alcohols, in the absence of water, were also investigated.

Figure S1 shows the influence of oxygenate addition the production of heptanal and 2-otanone. It is apparent that the addition of the alcohol to water increase the production of these longer chained oxygenates. Comparing the pure alcohol solvents to the mixed water/alcohol systems the quantity of 2-octanol produced is relatively unchanged, but the mixed systems produce significantly more heptanal. In the case of methanol/water ~4 mmol l^{-1} heptanal is formed. The higher yields of heptanol when both water and an alcohol are present may be associated with improved hydrogen-transfer from the alcohol in the presence of water, while the higher yields in methanol *cf.* 2-propanol are likely related to its greater efficacy as a hydrogen donor as previously reported by Xiang *et al.* (*App. Catal. A: Gen.* 2010, 375, 289–294.).

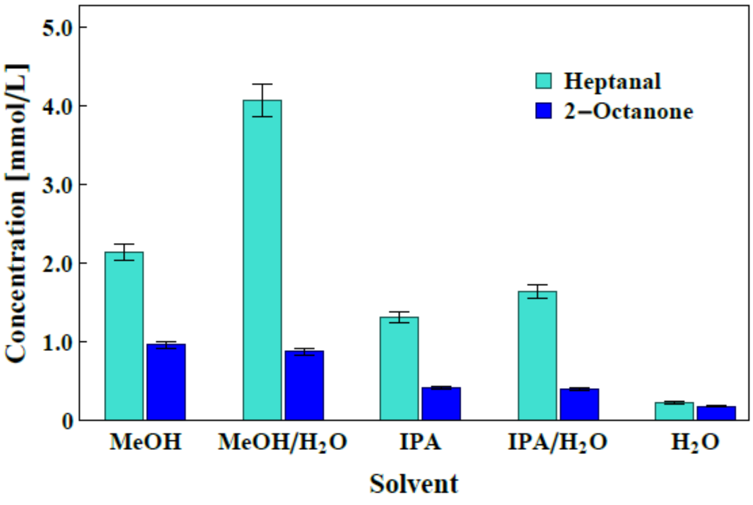

Figure S1. Influence of methanol and 2-propanol (denoted as IPA) on production of heptanal and 2-octanol in the hydrothermal conversion of CO_2. Reaction conditions: T = 300˚C, CO_2:H_2O mole ratio = 0.26, t = 4 h, 0.56 g catalyst.

In addition to heptanal and 2-octanone, reaction systems containing alcohols also produced heavier species with longer retention times in GCMS analysis when compared to the water-based reaction media (Figure S2). These longer-chained species have not been further characterised or identified. This increased production of high mass species occurs despite a lower conversion of CO_2 in the pure alcohol systems *cf.* water. The CO_2 conversions achieved were: water – 6.7%; water/methanol – 13.8%; methanol – 2.5%; water/2-propanol – 1.1%; 2-propanol – 2.7%. This suggests that a portion of the organic products were formed through direct conversion of the alcohols, with these providing the carbon source rather than exclusively CO_2.

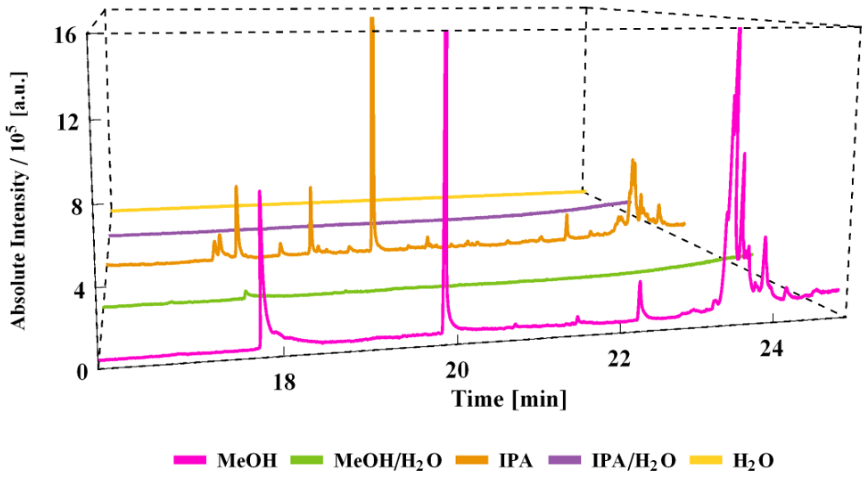

Figure S2. GCMS chromatograms of long retention time species obtained in water, methanol and 2-propanol (denoted as IPA) and mixed solvents in the hydrothermal conversion of CO_2. Reaction conditions: T = 300˚C, CO_2:H_2O mole ratio = 0.26, t = 4 h, 0.56 g catalyst.

In order to confirm that the alcohols can directly convert to longer-chained species under these conditions, the reaction involving methanol as the solvent was conducted using He in place of CO_2 (Figure S3). These data clearly show that high retention time products can be synthesised directly from short-chained organic species.

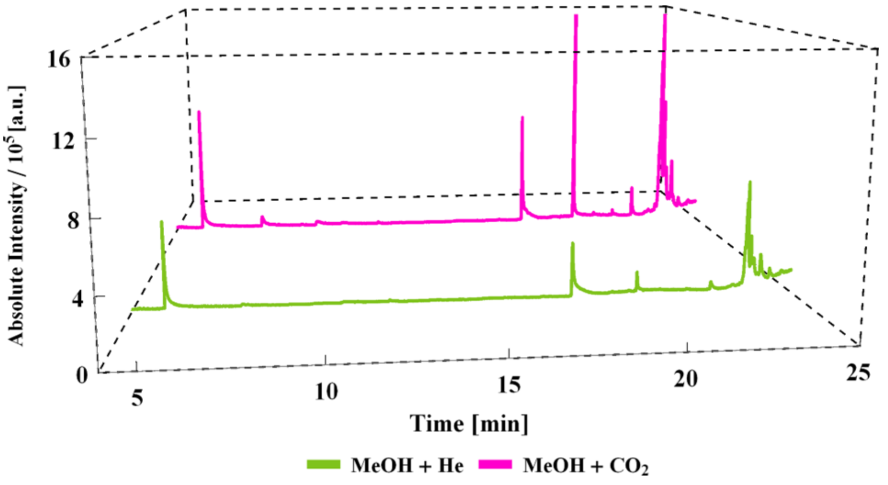

Figure S3. GCMS chromatograms of long retention time species obtained in methanol under both He and CO_2 atmosphere. Reaction conditions: T = 300˚C, CO_2/He:H_2O mole ratio = 0.26, t = 4 h, 0.56 g catalyst.

That organic species react directly under hydrothermal reaction conditions is further supported by complementary studies on biomass liquefaction. The combined liquefaction of biomass and CO_2 conversion – using sodium bicarbonate as a CO_2 source - has been studied under conditions similar to the present work by Andérez-Fernández *et al.*, (*Green Chem.*, submitted). Production of organic acids is observed even in the absence of sodium bicarbonate indicating the potential for transformations of organic species to occur directly under hydothermal conditions.

These data may suggest that there are in fact a number of parallel reaction mechanisms operating: the initial synthesis of short-chained species and their subsequent direct transformation not involving CO_2 and other mechanisms such as the aldol mechanism described in Section 3.4. These additional parallel reaction pathways corresponding hinder the ability to draw meaningful mechanistic conclusions on the role of these species on CO_2 conversion but shed valuable insights into the range of reactions occurring and the complexity of this reaction system. The conversion of methanol to higher oxygenates may occur *via* the condensation of intermediates such as formaldehyde and formyl rather than via the decomposition of methanol into CO and H_2 followed by a higher-alcohol synthesis (HAS) route. Therefore, in this case, CO_2 may promote the formation of the reaction intermediates, and thus, *via* subsequent condensation, enhance the production of higher oxygenates.
